# Implementation of dihydropyrimidine dehydrogenase deficiency testing in Europe

**DOI:** 10.1016/j.esmoop.2023.101197

**Published:** 2023-03-28

**Authors:** M. de With, A. Sadlon, E. Cecchin, V. Haufroid, F. Thomas, M. Joerger, R.H.N. van Schaik, R.H.J. Mathijssen, C.R. Largiadèr, ‘The Working Group on the Implementation of DPD-deficiency Testing in Europe’

**Affiliations:** 1 Department of Medical Oncology, Erasmus MC Cancer Institute, Rotterdam, the Netherlands; 2 Department of Clinical Chemistry, Erasmus University Medical Center, Rotterdam, the Netherlands; 3 Department of Clinical Chemistry, Inselspital, Bern University Hospital & University of Bern, INO F, Bern, Switzerland; 4 Department Experimental and Clinical Pharmacology Unit, Centro di Riferimento Oncologico di Aviano (CRO), IRCCS, Aviano, Italy; 5 Louvain Center for Toxicology and Applied Pharmacology (LTAP), Institut de Recherche Expérimentale et Clinique, UCLouvain, Brussels, Belgium; 6 Department of Clinical Chemistry, Cliniques Universitaires Saint-Luc, Brussels, Belgium; 7 Institut Claudius Regaud, IUCT-Oncopole and CRCT, University of Toulouse, Inserm, Toulouse, France; 8 Department of Internal Medicine, Klinik für Medizinische Onkologie & Hämatologie, Kantonsspital, St.Gallen, Switzerland

**Keywords:** DPD deficiency, fluoropyrimidine, *DPYD*, pre-therapeutic testing, implementation survey, toxicity

## Abstract

**Background:**

The main cause for fluoropyrimidine-related toxicity is deficiency of the metabolizing enzyme dihydropyrimidine dehydrogenase (DPD). In 2020, the European Medicines Agency (EMA) recommended two methods for pre-treatment DPD deficiency testing in clinical practice: phenotyping using endogenous uracil concentration or genotyping for *DPYD* risk variant alleles. This study assessed the DPD testing implementation status in Europe before (2019) and after (2021) the release of the EMA recommendations.

**Methods:**

The survey was conducted from 16 March 2022 to 31 July 2022. An electronic form with seven closed and three open questions was e-mailed to 251 professionals with DPD testing expertise of 34 European countries. A descriptive analysis was conducted.

**Results:**

We received 79 responses (31%) from 23 countries. Following publication of the EMA recommendations, 87% and 75% of the countries reported an increase in the amount of genotype and phenotype testing, respectively. Implementation of novel local guidelines was reported by 21 responders (27%). Countries reporting reimbursement of both tests increased in 2021, and only four (18%) countries reported no coverage for any testing type. In 2019, major implementation drivers were ‘retrospective assessment of fluoropyrimidine-related toxicity’ (39%), and in 2021, testing was driven by ‘publication of guidelines’ (40%). Although the major hurdles remained the same after EMA recommendations—‘lack of reimbursement’ (26%; 2019 versus 15%; 2021) and ‘lack of recognizing the clinical relevance by medical oncologists’ (25%; 2019 versus 8%; 2021)—the percentage of specialists citing these decreased. Following EMA recommendations, 25% of responders reported no hurdles at all in the adoption of the new testing practice in the clinics.

**Conclusions:**

The EMA recommendations have supported the implementation of DPD deficiency testing in Europe. Key factors for successful implementation were test reimbursement and clear clinical guidelines. Further efforts to improve the oncologists’ awareness of the clinical relevance of DPD testing in clinical practice are needed.

## Highlights

• EMA recommendations regarding pre-treatment DPD testing have supported the widespread implementation of testing in Europe.
• Key factors for successful implementation of DPD testing are test reimbursement and existence of clear clinical guidelines.
• Knowledge about the clinical applicability of pharmacogenetics among clinicians should be further improved.

## Introduction

Fluoropyrimidines, which include 5-fluorouracil (5-FU) and capecitabine, are important components for the treatment of a wide range of solid tumors, including colorectal, gastric, pancreatic, esophageal, breast, and head and neck cancer.^1^^,^^2^ Despite their frequent use in oncological clinical practice, fluoropyrimidines are associated with toxicities, which may lead to treatment interruption or discontinuation, hospitalization, or even death.^3^, ^4^, ^5^, ^6^, ^7^, ^8^

Dihydropyrimidine dehydrogenase (DPD) deficiency is the most important risk factor for developing fluoropyrimidine-related adverse events. DPD is the main metabolizing enzyme of 5-FU in the liver. In DPD-deficient patients, a shift towards the formation of active metabolites is observed.^9^^,^^10^ Different phenotyping and genotyping strategies for the identification of DPD-deficient patients have been developed.^11^ In 2020, the European Medicines Agency (EMA) recommended one of two methods for DPD deficiency testing in clinical practice: phenotyping by measuring the pre-treatment uracil concentration in the plasma or genotyping for the presence of certain *DPYD* risk variant alleles, namely c.1905+1G>A (rs3918290, *DPYD*^∗^2A, IVS14+1G>A), c.1679T>G (rs55886062, *DPYD*^∗^13, I560S), c.2846A>T (rs67376798, D949V), and c.1129–5923C>G [rs75017182; HapB3 or its tagging single nucleotide polymorphism (SNP) c.1236G>A; rs56038477].^12^, ^13^, ^14^ In patients identified as DPD deficient, the starting dose of fluoropyrimidines should then be reduced.^12^

As endogenous uracil is transformed into dihydrouracil by DPD, an elevated pre-treatment uracil concentration in plasma (>14 or 16 ng/ml) is predictive for severe fluoropyrimidine-related adverse events.^15^, ^16^, ^17^ Genotyping for certain variants in the gene coding for DPD, *DPYD*, was also found to be predictive for DPD deficiency and therefore increased risk of severe adverse events.^3^^,^^14^^,^^18^^,^^19^ Pre-therapeutic genotyping to reduce toxicity has been studied prospectively, and pre-treatment screening followed by a dose reduction in *DPYD* variant allele carriers was found to improve patient safety.^14^ Currently, the genotyping of the aforementioned four SNPs is advised.^13^^,^^14^ These include important, but not all, clinically relevant mutations. Recently, for instance, the relevance of the *DPYD*^∗^7 mutation has been published.^20^

Despite the strong evidence supporting the value of testing for DPD deficiency and inclusion of the EMA recommendations in the drug labels,^1^^,^^2^ DPD testing is not yet used worldwide.^21^^,^^22^ Recently, a survey conducted among 325 US medical oncologists reported that only 17 out of 59 respondents strongly agreed with the usefulness of DPD testing.^22^ Koo et al*.*^22^ suggested that lack of clinical practice guidelines recommending DPD testing was one of the most important factors deterring oncologists from prescribing *DPYD* testing.

In Europe, the implementation of DPD testing seems to be ahead of the USA given the high number of national and regional guidelines developed. There are, however, still differences between European guidelines and clinical implementation practice.^23^ Therefore, the aim of our current study was to assess the implementation status of DPD phenotyping and *DPYD* genotyping in Europe before (2019) and after (2021) the release of the EMA recommendations in order to identify key factors preventing or enabling the successful implementation of DPD deficiency testing.

## Methods

### Study population

The survey aimed at investigating the state of DPD phenotyping and genotyping implementation in Europe in 2019 and 2021 (i.e. the years just before and after the publication of the EMA recommendations). Inclusion criteria identified any professional with expertise in DPD testing in Europe (i.e. either scientific expertise or as test provider). We applied a multilevel strategy to identify potential contacts. First, we identified participants through professional connections, past publications on the topic, networks of experts [working groups for the Clinical Pharmacogenetics Implementation Consortium (CPIC) and the Golden Helix Foundation, an international non-profit organization with a focus on translational research and projects including projects aimed at promoting pharmacogenetic testing in Europe]. Second, we searched for DPD testing providers in Orphanet (directory of expert centers, search term: dihydropyrimidine dehydrogenase deficiency, selected: all countries), a dedicated directory of laboratory services offering DPD testing in Europe.^24^ Third, further contacts were identified through research on the internet with terms ‘*DPYD*’, ‘DPD’, and ‘test’ combined with the respective European countries, using the official language of the respective country. Finally, we requested that all study responders provide us contact information of additional experts or DPD testing providers to include in our survey.

### Data collection

The survey was conducted from 16 March 2022 to 31 July 2022 using a PDF form created in Adobe Acrobat (Supplementary Appendix 2, available at https://doi.org/10.1016/j.esmoop.2023.101197). One personal reminder was sent 1 week before the end of the survey. A total of 10 questions were asked (mix of open and closed questions). For each question, responders had the choice to provide additional comments in the appropriate field. Responders’ profiles (i.e. test provider versus test performer, primary versus secondary versus tertiary level institution) were also collected.

### Data analysis

We conducted a descriptive analysis of the responses. Survey answers were assessed by two clinical investigators and, if necessary, discussed with the study team. After calculating differences in the number of genotyping and phenotyping tests before and after the EMA recommendation for each responding center, we calculated the median difference per country. For questions related to quantitative estimations of regional and national implementation, the median value for each country was selected to account for outliers. Open questions were transformed into categorical variables. Considering the large heterogeneity in cost reimbursements and conditions reported, we created five categories to represent cost reimbursements: ‘no’ (i.e. service for self-payers only), ‘yes—no conditions’, ‘yes with conditions’, ‘yes—but restricted panel’, and ‘regional differences’. Advanced graphical visualization was conducted using the ggplot2, maps, and mapproj packages in R version 4.1.2.

## Results

### Responders

**Figure 1:**
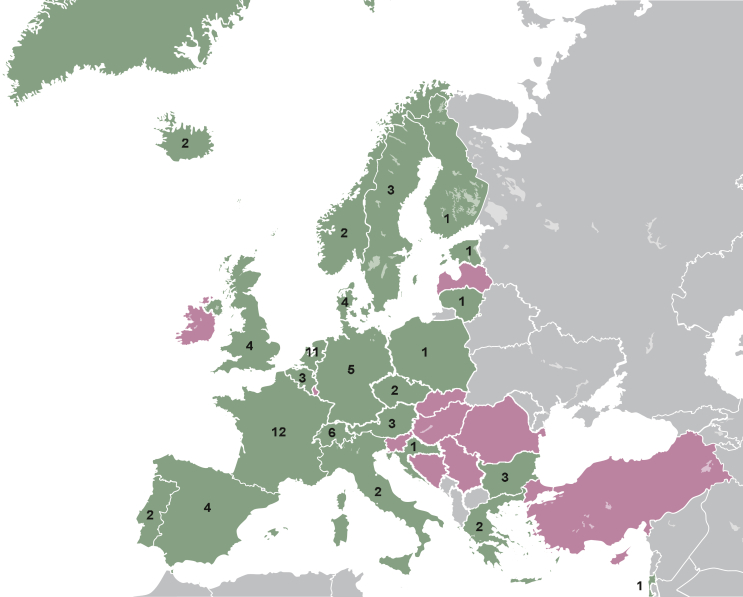
**Overview of contacted experts in Europe.** An overview of the countries with responders (green) and with contacted experts but without response (purple). The numbers show the number of responding centers per country.

**Figure 2:**
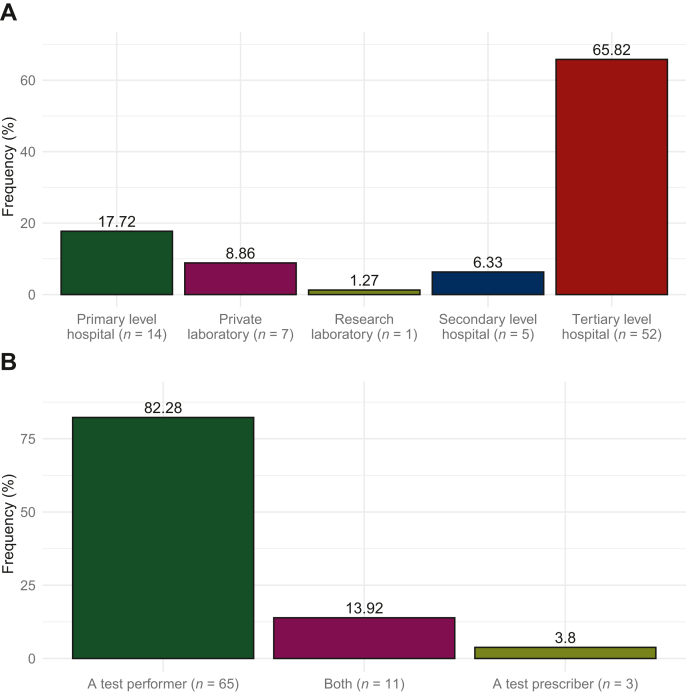
**Responders characteristics.** The responder characteristics: institution type (A), whether responders were test performers or test prescribers (B).

In total, 251 experts in 36 European countries were contacted by e-mail. After 1 July 2022, 79 positive responses from 23 countries were received, resulting in a response rate of 31%. In Figure 1, an overview of the countries with and without responders is depicted. The majority of responders (66%) worked in a tertiary level hospital (i.e. university hospital), and most responders were test performers (82%) (Figure 2A and B).

### Changes after EMA recommendations

**Figure 3:**
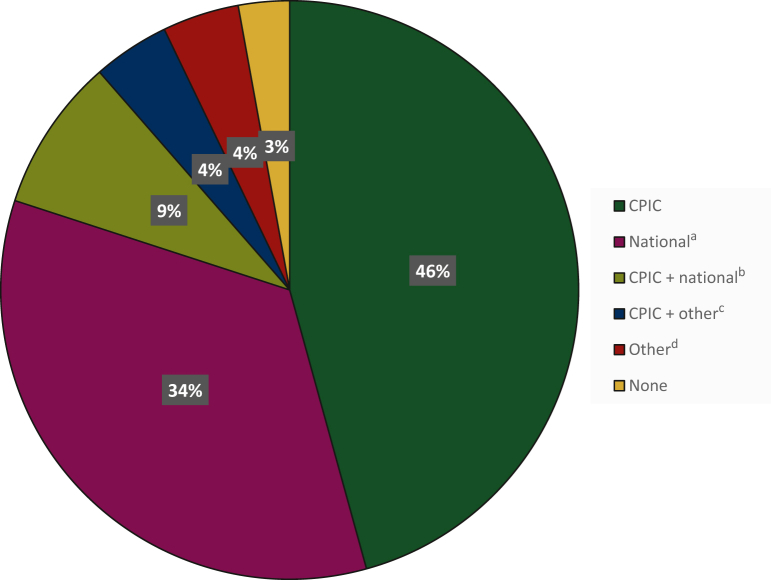
**Currently used guidelines in Europe.** The used guidelines in Europe, after the EMA recommendations are shown, by percentage of responders. CPIC, Clinical Pharmacogenetics Implementation Consortium. ^a^In three cases, a national guideline from another country was used. ^b^In two cases, a national guideline from another country was used. ^c^Institutional guideline, EMA recommendation, publication Henricks et al., *Lancet Oncology*, 2018. ^d^EMA recommendation, publication Henricks et al., *Lancet Oncology*, 2018, ESMO guideline.

According to 21 responders (30%), publication of the EMA recommendations resulted in the development of national, regional, or institutional guidelines in which advice regarding DPD deficiency testing was given. The most commonly used guideline in Europe is the CPIC guideline (59%); in some centers this guideline is combined with a national or institutional guideline. Of note, the CPIC guideline only provides recommendations on how to interpret the results and how to translate them into clinical actions. Thirty-four percent of the responders answered that a national guideline has been developed and is currently in use; in four cases, a national guideline from a different country was applied. An overview of the currently used guidelines is depicted in Figure 3.

The four aforementioned recommended SNPs were much more covered in 2021 than in 2019 (93% versus 60%; Supplementary Figure S1, available at https://doi.org/10.1016/j.esmoop.2023.101197). In 2019, 17% of the responders genotyped fewer than the four *DPYD* variants, and 10% of the responders genotyped for additional variants [i.e. c.2194G>A (*DPYD*^∗^6), c.299_302delTCAT (*DPYD*^∗^7), and additional variants because of whole genome sequencing; Supplementary Table S1, available at https://doi.org/10.1016/j.esmoop.2023.101197]. This shifted in 2021, when 6% of the responders genotyped fewer than the four recommended SNPs and 18% of the responders genotyped for additional variants. In 2021, only 1% of the responders did not carry out genotyping at all, compared with 22% in 2019.

Overall, after publication of the EMA recommendations, the total number of genotyping tests reported by the survey responders roughly doubled (from 25 641 in 2019 to 54 018 tests in 2021). A total of 20 countries (87%) reported an increase, whereas responding centers from three countries (Czech Republic, France, and Greece) mentioned a decrease in genotype testing (Supplementary Figure S2, available at https://doi.org/10.1016/j.esmoop.2023.101197). In the comment sections of the survey, the responders of these three countries gave oncologists’ lack of interest, lack of funding, and the COVID-19 pandemic as an explanation for this. Similarly, a rise in the use of phenotype testing was reported by the survey responders in a majority of countries (from 36 009 to 42 837 tests) with the exception of the responding centers from France [median (IQR: interquartile range) 2021-2019: −14% (−37%; 17%)] (Supplementary Figure S2, available at https://doi.org/10.1016/j.esmoop.2023.101197). Of note, the responders specified that 77% and 75% of the reported numbers in 2019 and 2021 were based on accurate data (i.e. not estimated test numbers).

### Drivers and hurdles for implementation of DPD deficiency testing

An overview of the drivers, stakeholders, and hurdles for implementation is shown in Supplementary Figure S3A-C, available at https://doi.org/10.1016/j.esmoop.2023.101197. In 2019, the main drivers of the implementation of DPD testing were the ‘retrospective assessment of severe fluoropyrimidine-related toxicity’ and therefore requests by medical oncologists (39%), followed by initiatives by leading laboratory professionals, pharmacologists, and (other) scientists in the field of *DPYD* deficiency testing (17%) (Supplementary Figure S3A, available at https://doi.org/10.1016/j.esmoop.2023.101197). We received similar answers when asked what the most important stakeholders were for implementation in 2019. These were medical oncologists (73%), followed by pharmacologists (8%) and national health authorities (4%) (Supplementary Figure S3B, available at https://doi.org/10.1016/j.esmoop.2023.101197). In 2019, the most important hurdles were ‘lack of reimbursement’ (26%), ‘lack of seeing the clinical relevance by oncologists’ (25%), and ‘lack of (a) clear national guideline(s)’ (15%) (Supplementary Figure S3C, available at https://doi.org/10.1016/j.esmoop.2023.101197).

After the publication of the EMA recommendations, the most important driver of the implementation of DPD testing changed to the ‘existence of the EMA guideline and other national guidelines’ (40%), followed by the ‘retrospective assessment of severe fluoropyrimidine-related toxicity’ (16%). The oncologist remained the major stakeholder for 39% of the responders. Interestingly, oncological societies became the second most important stakeholder for implementation (13%). The number of hurdles drastically changed over time, however, and in 2021, 25% of the responders reported no hurdles at all. The ‘lack of reimbursement’ was still mentioned as a hurdle in 15% of responses, and the ‘lack of seeing the clinical relevance by medical oncologists’ was reported in 8% of responses.

### Reimbursement of DPD deficiency tests

**Figure 4:**
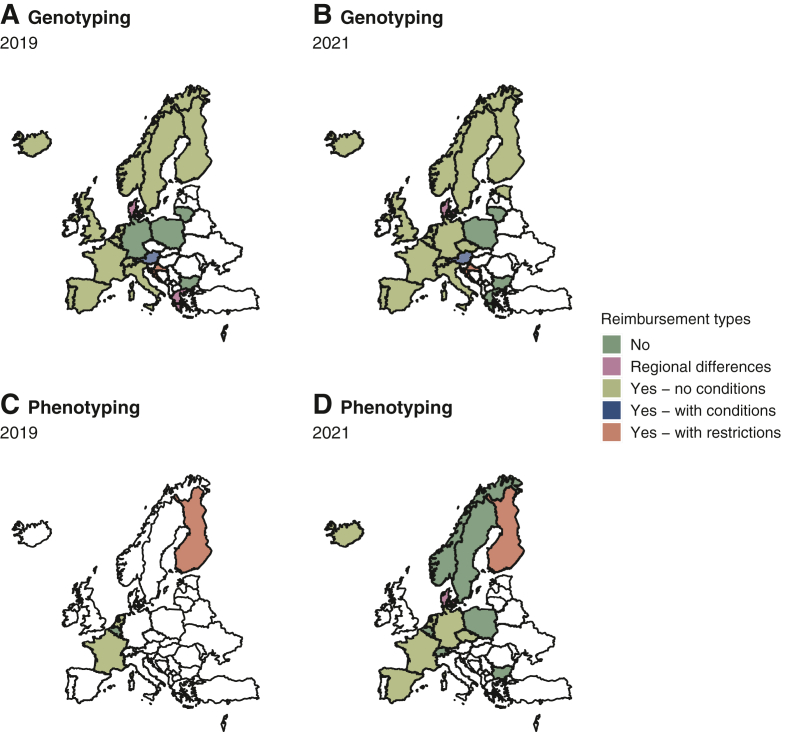
**Landscape of cost reimbursements for genotyping and phenotyping in 2019 and 2021.** Reimbursement types have been categorized into five groups (no, regional differences within a same country, yes—no conditions, yes—with conditions, and yes—with restrictions). The reimbursement type ‘yes—with conditions’ is defined by reimbursement of testing if certain conditions are met (for instance hospitalization, test prescriber is an oncologist) whereas the reimbursement type ‘yes—with restrictions’ defines reimbursement for some selected single nucleotide polymorphisms. White color represents nonresponding countries or countries not offering the test.

Reimbursement of both genotype and phenotype testing varied significantly across the countries, highlighting in part the different health care systems in Europe (Figure 4). Nevertheless, in 2019, more than half of the countries (55%) reported reimbursements for *DPYD* genotyping without any additional condition, a number which increased to 68% in 2021. Regional differences were noted in Denmark, whereas in Greece, a center reported reimbursement for genotyping in 2019 in the context of a project funded by the European Union. In Austria, the reimbursement of genotyping depended on the type of hospital and doctor prescribing the test, whereas in Israel, costs were reimbursed for inpatients in 2019 and then by the national health insurance in 2021. Of note, by 2021, neither genotyping nor phenotyping was reimbursed in four countries (Bulgaria, Greece, Lithuania, and Poland). In the countries carrying out phenotype tests, two countries (France and The Netherlands) reported coverage by the national/social health insurance in 2019, with the addition of Germany, Israel, and Spain in 2021 (Figure 4).

### Implementation of DPD deficiency testing

**Figure 5:**
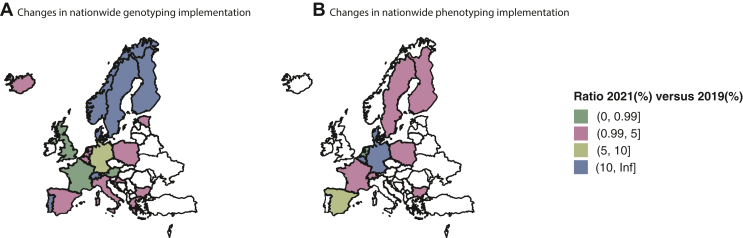
**Nationwide and regionwide implementation of genotyping and phenotyping.** (A) Fold change in nationwide genotyping rates between 2021 and 2019. (B) Fold change in nationwide phenotyping rates between 2021 and 2019.

Based on the survey, a nationwide increase of 76% was seen for genotyping after publication of the EMA recommendations (Figure 5A). Similarly, 63% of the responders reported a nationwide increase for phenotyping (Figure 5B).

The most frequently used genotyping method in both 2019 and 2021 was targeted genotyping (identification of the genetic variants of interest). Four centers used a combination of targeted genotyping followed by sequencing (identification of regions of the genome). For phenotyping, liquid chromatography mass spectrometry (LC-MS) was the most frequently used method in both 2019 and 2021. About one in four responders (23%) reported using DPD enzyme activity in peripheral blood mononuclear cells (PMBCs) for phenotyping; this number dropped to 10% in 2021. In parallel, an increase in the use of LC-MS was observed (46% in 2019 versus 67% in 2021). Overviews of the genotyping and phenotyping methods are shown in Supplementary Figure S4A and B, available at https://doi.org/10.1016/j.esmoop.2023.101197.

## Discussion

In this Europe-wide survey, we demonstrated how the 2020 EMA recommendations regarding DPD deficiency testing were followed by a noticeable increase in both phenotype and genotype testing. In some countries, such as France and The Netherlands, DPD deficiency testing was already observed before the release of the EMA recommendations; this was mainly promoted by large studies providing supportive evidence for pre-therapeutic DPD testing and by guidelines.^3^^,^^13^, ^14^, ^15^^,^^18^^,^^25^ These studies also provided the scientific foundation for the development of the EMA recommendations. As was highlighted by the respondents contacted in this survey, the EMA recommendations were paramount to overcoming some of the hurdles, which had so far hampered DPD deficiency testing implementation in various countries. Specifically, hurdles included lack of reimbursement, poor knowledge and consideration by oncologists, and absence of clear guidelines. For the latter, the EMA recommendations supported the development and implementation of several national, regional, and institutional guidelines in many European countries including Belgium,^26^ Denmark,^27^ England,^28^ Finland,^29^ Italy,^30^ Spain,^31^ and Switzerland,^32^ and a joint guideline for Switzerland, Germany, and Austria^33^ (Supplementary Appendix 3, available at https://doi.org/10.1016/j.esmoop.2023.101197).

Despite a general positive trend in testing in Europe, some differences are noted between the countries. For instance, genotype testing decreased in France, possibly as a consequence of the guidelines from the French health authority (Haute Autorité de la Santé) published in 2018 recommending DPD phenotyping over *DPYD* genotyping.^34^ Their report states that measuring uracil more directly determines the functional activity of the DPD enzyme, regardless of the genetic and non-genetic elements from which this activity arises.^34^ The possible impact of unknown *DPYD* variants and the functional consequences of a combination of *DPYD* variants are thus better predicted.^34^ At the same time, we observed an overall decrease in phenotyping in France between 2019 and 2021. This apparent decrease is explained by the fact that DPD phenotyping was already mandatory and massively carried out in 2019 in the laboratories participating in the survey. As suggested by responders from several countries, the COVID pandemic in 2020 modified DPD testing practices in their respective hospitals and may have contributed to the observed decrease. Regional differences are also observed, such as in The Netherlands, where a decrease in testing was reported for the Rotterdam center. This is explained by an increase in the number of laboratories carrying out DPD deficiency testing, which led to decreasing demands for the tertiary center; this pattern was also observed in France.

Our survey emphasizes how a lack of funding and cost reimbursements remains a critical hurdle for DPD deficiency testing implementation. This was emphasized by respondents from Greece, who noted decreased testing after funding ended. Whereas nearly two-thirds of the countries represented in our survey offer full coverage of the testing costs in 2021, four of the responding countries (Bulgaria, Greece, Lithuania, and Poland) are still facing a lack of reimbursements. Despite this, rising genotype testing numbers are noted in Bulgaria, Lithuania, and Poland in 2021, thereby highlighting how the EMA guidelines have also served as a major stepping stone for implementing DPD testing in these countries. In countries where cost coverage is limited, international collaborative research projects and initiatives (such as the Golden Helix Foundation) may help raise awareness of the importance of DPD testing at the national level. In this regard, we observed that countries with national guidelines were more likely to reimburse DPD deficiency testing. This may explain why phenotyping is reimbursed in fewer countries than genotyping, as the latter remains the recommended gold standard in several countries.

According to the responders, lack of knowledge of the clinical relevance of DPD deficiency testing among clinical oncologists was also an important hurdle for successful implementation. A study by Dressler et al*.*^35^ shows that most medical oncologists are not equipped with the most relevant pharmacogenetic knowledge and do not always see the clinical relevance of pharmacogenetic testing. Moreover, medical oncologists do not always understand how to interpret and apply pharmacogenetic results in clinical practice.^35^ A study by Begré et al.^36^ noticed that oncologists who were part of research collaborations related to DPD deficiency testing were ‘early adopters’ of DPD deficiency testing in clinical practice. This highlights how knowledge about clinical relevance impacts implementation of the test. Education on pharmacogenetics in clinical practice by (for example) clinical chemists, in combination with clear guidelines on how to treat patients with different genotypes, will improve implementation of DPD deficiency testing.

This is the first study examining the changes in DPD testing in Europe following the EMA recommendations in 2020. Moreover, compared with a similar survey conducted in the USA, our study shows a higher response rate of 31% (compared with 18% in the previously mentioned study). With its coverage of 23 countries, our study provides a valuable and reliable picture of the state of DPD deficiency testing in Europe. Our study, however, also has some limitations. First, most responders worked in a tertiary level hospital (66%), which may lead to an overestimation of the tests carried out because new guidelines are often initiated by academic hospitals before being implemented in smaller hospitals such as secondary or primary level hospitals. Second, test prescribers (18%) represented only a minority of the responders even though medical oncologists were a major driver for implementation of DPD testing in clinical practice. Furthermore, as shown in our survey, oncologists’ lack of knowledge of the clinical relevance of DPD deficiency testing in clinical practice constitutes a critical barrier for DPD deficiency testing implementation. Third, although we used an extensive strategy to contact as many experts as possible across Europe, information and insights from Eastern Europe countries were scarce. This may reflect a lack of implementation in these countries. In this regard, it is worth mentioning that we observed a high number of responders in France and The Netherlands, where implementation was established well before the EMA recommendations. Additionally, we focused on contacting professionals with DPD deficiency expertise because contacting other health professionals (e.g. nurses or patient organizations) would be much more challenging with respect to factors including communication methods and language barriers. Fourth, the COVID-19 pandemic affected testing in several centers. Finally, for some centers, the numbers provided were estimates of the number of tests, and the responses we depicted per country are not necessarily representative for the whole country.

### Conclusion

Implementation of DPD deficiency testing in Europe, by measuring pre-treatment plasma uracil concentration or by *DPYD* genotyping before initiation with fluoropyrimidine treatment, has markedly increased after publication of the 2020 EMA recommendations. Key factors for successful implementation are reimbursement of the specific tests and clear clinical guidelines. Stakeholders should be aware of the lack of knowledge about the clinical applicability of pharmacogenetics among clinicians.
