# Supplementary material for: Implementation of dihydropyrimidine dehydrogenase deficiency testing in Europe
Source: ESMO Open. 2023 Mar 28;8(2):101197. doi: 10.1016/j.esmoop.2023.101197 (PMC10163157; doi:10.1016/j.esmoop.2023.101197)
Supplement: Supplementary Appendix 1 [file mmc1.docx]

**Supplementary Appendix 1**

Members of The European Working Group on the Implementation of DPD deficiency testing in Europe

| Alarcan Hugo, CHRU de Tours, Tours, France, email: Hugo.Alarcan@univ-tours.fr |
| --- |
| Ambrodji Alisa, Department of Clinical Chemistry, Bern University Hospital, Switzerland, email: alisa.ambrodji@extern.insel.ch |
| Amstutz Ursula, Department of Clinical Chemistry, Bern University Hospital, Switzerland, email: ursula.amstutz@insel.ch |
| Arenas Hernandez Monica, Purine Research Lab. Synnovis, London, United Kingdom, email: [monica.arenas-hernandez@gstt.nhs.uk](mailto:monica.arenas-hernandez@gstt.nhs.uk) |
| Axelsson Magnus, Sahlgrenska University Hospital, Goeteborg, Sweden, email: magnus.axelsson@vgregion.se |
| Baars Arnold, Ziekenhuis Gelderse Vallei, Ede, Netherlands, email: baarsa@zgv.nl |
| Begre Ursina, Department of Clinical Chemistry, Bern University Hospital, Switzerland, email: ursina.begre@students.unibe.ch |
| Bjanes Tormod Karlsen, Helse Bergen HF, Bergen, Norway, email: tormod.karlsen.bjanes@helse-bergen.no |
| Boige Valerie, Gustave Roussy, Paris, France, email: valerie.boige@gustaveroussy.fr |
| Boyer Jean Christophe, University Hospital Nimes, Nimes, France, email: jean.christophe.boyer@chu-nimes.fr |
| Bozina Nada, University Hospital Centre Zagreb, Zagreb, Croatia, email: nada.bozina@mef.hr |
| Brueggemann Monika, Labor fuer haematologische Spezialdiagnostik, University Hospital Schleswig-Holstein, Campus Kiel, Germany, email: m.brueggemann@med2.uni-kiel.de |
| Brufau Dones Gemma, Albert Schweitzer hospital - Result Laboratory, Dordrecht, Netherlands, email: g.brufaudones@asz.nl |
| Brunet Mercedes, Clinic Barcelona, University of Barcelona, Spain, email: mbrunet@clinic.cat |
| Cado Sylvie, CERBA Laboratory , Cergy-Pontoise, France, email: scado@lab-cerba.com |
| Carracedo Angel, Fundacion Publica Galega de Medicina Xenomica (IDIS) and CIBERER- CIMUS, University of Santiago de Compostela Santiago de Compostela, Spain, email: angel.carracedo@usc.es |
| Cats Annemieke, Netherlands Cancer Institute - Antoni van Leeuwenhoek, Amsterdam, Netherlands, email: a.cats@nki.nl |
| Ciccolini Joseph, University Hospital of Marseille, Marseille, France, email: ciccolini.joseph@gmail.com |
| Claes Kathleen, UZ Gent, Gent, Belgium, email: kathleen.claes@uzgent.be |
| Damkier Per, Odense University Hospital, Odense, Denmark, email: pdamkier@health.sdu.dk |
| Danesi Romano, Unit of Clinical Pharmacology and Pharmacogenetics, Department of Clinical and Experimental Medicine, University of Pisa, Pisa, Italy, email: romano.danesi@unipi.it |
| Deenen Maarten, Catharina Hospital , Leiden, Netherlands, email: maarten.deenen@catharinaziekenhuis.nl |
| Deiman Birgit, Catharina Hospital Eindhoven, Eindhoven, Netherlands, email: birgit.deiman@catharinaziekenhuis.nl |
| Del Re Marzia, Unit of Clinical Pharmacology and Pharmacogenetics, Department of Clinical and Experimental Medicine, University of Pisa, Pisa, Italy, email: marzia.delre@unipi.it |
| Delacour Herve, Begin Military Hospital, Paris, France, email: herve.delacour@intradef.gouv.fr |
| Deneer Vera, University Medical Center Utrecht, Utrecht, Netherlands, email: V.H.M.Deneer@umcutrecht.nl |
| Dideberg Vinciane, CHU Liege, Liege, Belgium, email: vinciane.dideberg@chuliege.be |
| Efrati Edna, Rambam Medical Center, Haifa, Israel, email: e_efrati@rambam.health.gov.il |
| Etienne-Grimaldi Marie Christine, Centre Antoine Lacassagne, Nice, France, email: marie-christine.etienne-grimaldi@nice.unicancer.fr |
| Findeisen Peter, Labor Limbach, Heidelberg, Germany, email: peter.findeisen@labor-limbach.de |
| Ganoci Lana, University Hospital Centre Zagreb, Zagreb, Croatia, email: lana.ganoci@gmail.com |
| Geboes Karen, UZ Gent, Gent, Belgium, email: karen.geboes@uzgent.be |
| Grudeva Janet, Medical University Plovdiv, Plovdiv, Bulgaria, email: dr_grudeva@yahoo.com |
| Guchelaar Henk-Jan, Leiden University Medical Center, Leiden, Netherlands, email: h.j.guchelaar@lumc.nl |
| Haraldsdottir Sigurdis, Landspitali, Reykjavik, Iceland, email: sigurdish@landspitali.is |
| Haverkamp Thomas, ÜBAG MVZ Dr. Eberhard & Partner, Dortmund, Germany, email: Haverkamp@labmed.de |
| Hennart Benjamin, CHU Lille, Lille, France, email: benjamin.hennart@chru-lille.fr |
| Ivanov Hristo, Medical University Plovdiv, Plovdiv, Bulgaria, email: doctorhristoivanov@yahoo.com |
| Jennings Barbara, University East Anglia, Norwich, United Kingdom, email: B.Jennings@uea.ac.uk |
| Jonsson Jon Johannes, Landspitali, Reykjavik, Iceland, email: jonjj@landspitali.is |
| Kahre Tiina, Tartu University Hospital, Genetics and Personalized Medicine Clinic; Tartu University, Department of Clinical Genetics, Tartu, Estonia, email: tiina.kahre@kliinikum.ee |
| Kaneva Radka, Medical University of Sofia, Sofia, Bulgaria, email: kaneva@mmcbg.org |
| Kerr David, University of Oxford, Oxford, United Kingdom, email: david.kerr@ndcls.ox.ac.uk |
| Kleibl Zdenek, Institute of Medical Biochemistry and Laboratory Diagnostics, First Faculty of Medicine, Charles University and General University Hospital in Prague, Prague, Czech Republic, email: zdekleje@lf1.cuni.cz |
| Kleiblova Petra, Institute of Medical Biochemistry and Laboratory Diagnostics, First Faculty of Medicine, Charles University and General University Hospital in Prague, Prague, Czech Republic, email: pekleje@lf1.cuni.cz |
| Knikman Jonathan, Netherlands Cancer Institute - Antoni van Leeuwenhoek, Amsterdam, Netherlands, email: j.knikman@nki.nl |
| LLerena Adrián, Extremadura University Medical School, Badajoz University Hospital, Badajoz, Spain, email: adrian.llerena@salud-juntaex.es |
| Lopez-Fernandez Luis Andrés, Hospital General Universitario Gregorio Marañon, Instituto de Investigación Sanitaria Gregorio Marañón, Madrid, Spain, email: luis.lopez@iisgm.com |
| Loriot Marie Anne, Assistance Publique Hopitaux de Paris; European Georges-Pompidou hospital, department of clinical chemistry; University of Paris Cite, Paris, France, email: marie-anne.loriot@aphp.fr |
| Manolopoulos Vangelis, University Hospital of Alexandroupolis & Democritus University of Thrace Medical School, Alexandroupolis, Greece, email: emanolop@med.duth.gr |
| Maring Jan Gerard, Isala Hospital Zwolle, Zwolle, Netherlands, email: j.g.maring@isala.nl |
| Maronas Olalla, Fundacion Publica Galega de Medicina Xenomica (IDIS) and CIBERER- CIMUS, University of Santiago de Compostela Santiago de Compostela, Spain, email: olalla.maronas@usc.es |
| Marschon Renate, Labor fuer molekulargenetische Diagnostik (LMGD), Linz, Austria, email: Renate.Marschon@ordensklinikum.at |
| Meier Stephanie, University Hospital Basel, Basel, Switzerland, email: stephanie.meier@usb.ch |
| Michaud Joelle, Gene Predictis, Lausanne, Switzerland, email: jam@genepredictis.com |
| Moehlendick Birte, University Hospital Essen, Essen, Germany, email: Birte.Moehlendick@uk-essen.de |
| Nissen Peter, Aarhus University Hospital, Aarhus, Denmark, email: peteniss@rm.dk |
| Nygren Peter, Uppsala University, Uppsala, Sweden, email: peter.nygren@igp.uu.se |
| Oberkofler Hannes, Paracelsus Medical School Salzburg, Salzburg, Austria, email: H.Oberkofler@salk.at |
| Ottosson Jesper, Sahlgrenska University Hospital, Goeteborg, Sweden, email: jesper.ottosson@vgregion.se |
| Palles Claire, University of Birmingham, Birmingham, United Kingdom, email: c.palles@bham.ac.uk |
| Parejo Sarah, Dr Risch AG, Bern, Switzerland, email: sarah.parejo@risch.ch |
| Patrinos George P., University of Patras, Patras, Greece & United Arab Emirates University, Al-Ain, Abu Dhabi, UAE, email: gpatrinos@upatras.gr |
| Plomgaard Peter, Rigshospitalet, Copenhagen, Denmark, email: Peter.Plomgaard@regionh.dk |
| Popov Veselin, Medical University Plovdiv, Plovdiv, Bulgaria, email: dr.v_popov@yahoo.com |
| Raycheva Gabriela, Medical University Plovdiv, Plovdiv, Bulgaria, email: dr.graycheva@gmail.com |
| Renner Wilfried, Medical University of Graz, Graz, Austria, email: [wilfried.renner@medunigraz.at](mailto:wilfried.renner@medunigraz.at) |
| Rezai Keyvan, Institut Curie, Paris, France, email: keyvan.rezai@curie.fr |
| Rossing Maria, Rigshospitalet, Center for Genomic Medicine, Rigshospitalet, Copenhagen University Hospital, Copenhagen, Denmark, email: caroline.maria.rossing@regionh.dk |
| Royer Bernard, CHU Besancon, Besancon, France, email: broyer@chu-besancon.fr |
| Saarenheimo Jatta, Vasa Central Hospital, Vasa, Finland, email: Jatta.saarenheimo@fimlab.fi |
| Sabaliauskaite Rasa, National Cancer Institute, Vilnius, Lithuania, email: rasa.sabaliauskaite@nvi.lt |
| Samaan Simon, CERBA Laboratory , Cergy-Pontoise, France, email: simon.samaan@lab-cerba.com |
| Schwab Matthias, Dr Margarete Fischer-Bosch-Institute of Clinical Pharmacology, Stuttgart, Germany, and Department of Clinical Pharmacology, University Hospital Tübingen, Tübingen, Germany email: matthias.schwab@ikp-stuttgart.de |
| Silveira Catarina, GenoMed, SA, Lisbon, Portugal, email: csilveira@medicina.ulisboa.pt |
| Simicevic Livija, University Hospital Centre Zagreb, Zagreb, Croatia, email: lsimicev@kbc-zagreb.hr |
| Skogstad Tuv Silja, Oslo University Hospital, Oslo, Norway, email: silsko@ous-hf.no |
| Soucek Pavel, Charles University, Faculty of Medicine in Pilsen, Pilsen, Czech Republic, email: pavel.soucek@szu.cz |
| Spanaus Katharina, University Hospital Zurich, Zurich, Switzerland, email: katharina.spanaus@usz.ch |
| Stepman Hedwig, UZ Gent, Gent, Belgium, email: hedwig.stepman@uzgent.be |
| Sulzyc-Bielicka Violetta, Pomeranian Medical University, Szczecin, Poland, email: violabielicka@icloud.com |
| Svinarov Dobrin, Alexander Hospital, Medical University of Sofia, Sofia, Bulgaria, email: dsvinarov@yahoo.com |
| Swen Jesse, Leiden University Medical Center, Leiden, Netherlands, email: j.j.swen@lumc.nl |
| Teixeira Manuel, Portuguese Institute of Oncology of Porto (IPO Porto), Porto, Portugal, email: manuel.teixeira@ipoporto.min-saude.pt |
| van den Broek Daan, Netherlands Cancer Institute - Antoni van Leeuwenhoek, Amsterdam, Netherlands, email: da.vd.broek@nki.nl |
| van Zanden Jelmer, Certe, Groningen, Netherlands, email: j.vanzanden@certe.nl |
| Vetter Marcus, Kantonsspital Baselland, Liestal, Switzerland, email: marcus.vetter@ksbl.ch |
| Vorland Marta, Helse Bergen HF, Bergen, Norway, email: marta.vorland@helse-bergen.no |
| Wadelius Mia, Uppsala University Hospital, Uppsala, Sweden, email: Mia.Wadelius@medsci.uu.se |
