# Supplementary material for: Implementation of dihydropyrimidine dehydrogenase deficiency testing in Europe
Source: ESMO Open. 2023 Mar 28;8(2):101197. doi: 10.1016/j.esmoop.2023.101197 (PMC10163157; doi:10.1016/j.esmoop.2023.101197)
Supplement: Supplementary Appendix 2 [file mmc2.pdf]

Erasmus MC University Medical Center Rotterdam | Kantonsspital St.Gallen | INSELSPITAL UNIVERSITÄTSSPITAL BERN HÔPITAL UNIVERSITAIRE DE BERNE

SURVEY ON THE IMPLEMENTATION OF THE DPD DEFICIENCY TESTING IN EUROPE

On 30 April 2020,
the **European Medicines Agency** recommended
1) "that patients should be tested for the lack of the enzyme dihydropyrimidine dehydrogenase (DPD) before starting cancer treatment with fluorouracil given by injection or infusion (drip) or with the related medicines, capecitabine and tegafur".
2) "patients can be tested for DPD deficiency by measuring the level of uracil (a substance broken down by DPD) in the blood, or by checking for the presence of certain mutations (changes) in the gene for DPD (*DPYD*)".

Link

The **aim of this 10 questions survey is to assess the impact of the EMA recommendations on the implementation of *DPYD*-testing or DPD-phenotyping in European countries by comparing the situation in 2019 (before the EMA recommendations) and in 2021 (after the EMA recommendations)**. In particular, the survey also aims at identifying potential key factors preventing or enabling successful implementation of pharmacogenetic testing by comparing the implementation histories of *DPYD*-testing between different countries.

Please note! If you think some changes in *DPYD*-testing and DPD-phenotyping practices between 2019 and 2021 do not relate to or have not been triggered by the EMA recommendations in your country or at your center, provide the respective information as additional comments where appropriate.

QUESTION 1 of 10
IMPLEMENTATION OF *DPYD* TESTING OR DPD PHENOTYPING

Are you carrying out pharmacogenetic testing and/ or phenotyping test for *DPYD* at your centre?

in 2019?

	Yes	No	Method
Genotyping:	◉	○	
Phenotyping:	○	◉	

In 2021?

	Yes	No	Method
Genotyping:	◉	○	
Phenotyping:	○	◉	

Additional comments

QUESTION 2 of 10
TRENDS IN *DPYD* TESTING OR DPD PHENOTYPING

ABBREVIATIONS
R: retrospective, i.e. prescribed after the therapy start
P: prospective, i.e. prescribed before the therapy start
U: unknown, i.e. when it is unclear whether the test was undertaken before or after therapy start

How many tests did you perform per year at your centre?

in 2019?

Genotyping:

a) number of tests performed ____

this number is: *accurate* ◯ *estimated* ◯

b) proportions of retrospective tests and prospective tests, e.g. 20% R vs. 80% P:

estimated number: ____ % R vs ____ %P

if available, accurate number: ____ % R vs ____ %P ____ %U

Phenotyping:

a) number of tests performed ____

this number is: *accurate* ◯ *estimated* ◯

b) proportions of retrospective tests and prospective tests, e.g. 20% R vs. 80% P:

estimated number: ____ % R vs ____ %P

if available, accurate number: ____ % R vs ____ %P ____ %U

in 2021?

Genotyping:

a) number of tests performed ____

this number is: *accurate* ◯ *estimated* ◯

b) proportions of retrospective tests and prospective tests, e.g. 20% R vs. 80% P:

estimated number: ____ % R vs ____ %P

if available, accurate number: ____ % R vs ____ %P ____ %U

Phenotyping:

a) number of tests performed ____

this number is: *accurate* ◯ *estimated* ◯

b) proportions of retrospective tests and prospective tests, e.g. 20% R vs. 80% P:

estimated number: ____ % R vs ____ %P

if available, accurate number: ____ % R vs ____ %P ____ %U

Additional comments

QUESTION 3 of 10
ANALYSIS REPORTS GUIDELINES

a) **Please list the guideline(s) to which your analysis reports are currently referring to** (e.g. CPIC® Guideline for Fluoropyrimidines and *DPYD* – CPIC).

Link

b) **Was there any notable change in the guidelines following the publication of the EMA recommendations** (e.g. establishment of national guidelines)**?**

Additional comments

QUESTION 4 of 10
SNPs COVERED BY *DPYD* TESTING

Does your *DPYD* testing cover the four SNPs recommended by the CPIC guideline?

c.1129-5923C>A (rs75017182, c.1236G>A/HapB3)
c.2846A>T (rs67376798)
c.1679T>G (rs55886062,*13)
c.1905+1G>A (rs3918290,*2A)

in 2019?

Yes ○

No ○

Additional variants or missing variants:

in 2021?

Yes ○

No ○

Additional variants or missing variants:

Additional comments

QUESTION 5 of 10
COST REIMBURSEMENTS

Are the analysis costs reimbursed?

**in 2019?**	Genotyping:	Yes	No	By whom
	Phenotyping:	Yes	No	By whom
**in 2021?**	Genotyping:	Yes	No	By whom
	Phenotyping:	Yes	No	By whom

Additional comments

QUESTION 6 of 10
STAKEHOLDERS FOR IMPLEMENTATION

What were the most important stakeholders regarding the implementation of *DPYD*-testing or DPD-phenotyping in your country (e.g. diagnostic labs, oncological societies, patient organizations etc.)

please choose

in 2019?
Name the two most important stakeholders:

1. oncologists
2. oncologists

in 2021?
Name the two most important stakeholders:

1. oncologists
2. oncologists

Additional comments

QUESTION 7 of 10
HURDLES FOR IMPLEMENTATION

What was in your opinion the most important hurdle for the implementation of *DPYD*-testing or DPD-phenotyping in your country (e.g. lack of reimbursement)?

in 2019?

in 2021?

Additional comments

QUESTION 8 of 10
DRIVERS FOR IMPLEMENTATION

What was in your opinion the most important driver for the implementation of *DPYD*-testing or DPD-phenotyping in your country (e.g. requests by patients)?

in 2019?

in 2021?

Additional comments

QUESTION 9 of 10
NATIONWIDE IMPLEMENTATION

What is in your opinion the level of the implementation of *DPYD*-testing or DPD genotyping in your country (in percentage of the patients receiving FP-based chemotherapies/ year)?

**in 2019?**	Genotyping	estimated number (e.g. 10%)	
		if available, accurate number:	
	Phenotyping	estimated number (e.g. 10%)	
		if available, accurate number:	
**in 2021?**	Genotyping	estimated number (e.g. 10%)	
		if available, accurate number:	
	Phenotyping	estimated number (e.g. 10%)	
		if available, accurate number:	

Additional comments

QUESTION 10 of 10
REGIONAL DIFFERENCES IN IMPLEMENTATION

Were there regional differences in the implementation of *DPYD*-testing or DPD-phenotyping in your country?

**in 2019?**	Genotyping:	Yes ◯	No ◯
	Phenotyping:	Yes ◯	No ◯
**in 2021?**	Genotyping:	Yes ◯	No ◯
	Phenotyping:	Yes ◯	No ◯

Additional comments

PLEASE ADD ANY FURTHER COMMENTS IN THE BOX BELOW

Suggested expert to whom this questionnaire should also be sent to?

PLEASE FILL IN YOUR CONTACT DETAILS AND INSTITUTION DETAILS

First Name **Family Name**

Institution **Department**

About your institution

Type Tertiary level hospital ***Estimated number of individuals in the catchment area***

City **Country**

Email **You are** a test performer

LAST STEP: SAVE THE FILLED PDF FORM AND SEND IT TO OR CLICK THE BUTTON BELOW

angelique.sadlon@insel.ch

SEND

(in Adobe only)

We thank you in advance for your time!

The European Working Group on the Implementation of DPD deficiency testing in Europe

Prof. Markus Jörger MD PhD (Kantonsspital St Gallen, Switzerland), Prof. Carlo Largiadèr PhD (Inselspital Bern, Switzerland), Prof. Ron Mathijssen MD PhD (Erasmus Medical Centre Rotterdam, Netherlands), Prof. Ron van Schaik PhD (Erasmus Medical Centre Rotterdam, Netherlands), Mirjam de With MD (Erasmus University Rotterdam, Netherlands), Ursina Begré M Med (Inselspital Bern, Switzerland), Dr. Angélique Sadlon MD PhD (Inselspital Bern, Switzerland)
