# Supplementary material for: Implementation of dihydropyrimidine dehydrogenase deficiency testing in Europe
Source: ESMO Open. 2023 Mar 28;8(2):101197. doi: 10.1016/j.esmoop.2023.101197 (PMC10163157; doi:10.1016/j.esmoop.2023.101197)
Supplement: Supplementary Appendix 3 [file mmc3.docx]

**Supplementary Appendix 3: European guidelines regarding DPD deficiency testing**

European countries with a national guideline regarding DPD deficiency testing, followed by reference:

- Belgium^1^
- Denmark^2^
- England^3^
- Finland^4^
- Italy^5^
- Spain^6^
- The Netherlands^7^
- Switzerland^8^
- Joint guideline for Switzerland, Germany, and Austria^9^

1. Casneuf V, Borbath I, Van den Eynde M, Verheezen Y, Demey W, Verstraete AG, et al. Joint Belgian recommendation on screening for DPD-deficiency in patients treated with 5-FU, capecitabine (and tegafur). Acta Clin Belg. 2022;77(2):346-52.

2. Dansk Selskab For Klinisk Biokemi. Test for aktivitet af dihydropyrimidin dehydrogenase forud for behandling med 5-fluorouracil- (i.v.), capecitabin- og tegafurholdige præparater: Dansk Selskab For Klinisk Biokemi; 2021 [Available from: <https://dskb.dk/wp-content/uploads/2021/04/NationalkonsensusDPD-_17.03marts2021.pdf>.

3. National Health Service England. Clinical Commissioning Urgent Policy Statement Pharmacogenomic testing for DPYD polymorphisms with fluoropyrimidine therapies: National Health Service England; 2020 [updated 25-11-2020. Available from: <https://www.england.nhs.uk/wp-content/uploads/2020/11/1869-dpyd-policy-statement.pdf>.

4. terveysportti. Kolorektaalisyövän kansalliset hoitosuositukset 2022 [Available from: <https://www.terveysportti.fi/apps/ltk/article/hsu00007?toc=507>.

5. A cura del Gruppo di Lavoro di AIOM - SIF. Raccomandazioni per analisi farmacogenetiche: Associazione Italiana di Oncologia Medica; 2020 [Available from: <https://www.aiom.it/wp-content/uploads/2019/10/2019_Racc-analisi-farmacogenetiche_v26.3.2020.pdf>.

6. García-Alfonso P, Saiz-Rodríguez M, Mondéjar R, Salazar J, Páez D, Borobia AM, et al. Consensus of experts from the Spanish Pharmacogenetics and Pharmacogenomics Society and the Spanish Society of Medical Oncology for the genotyping of DPYD in cancer patients who are candidates for treatment with fluoropyrimidines. Clin Transl Oncol. 2022;24(3):483-94.

7. Lunenburg C, van der Wouden CH, Nijenhuis M, Crommentuijn-van Rhenen MH, de Boer-Veger NJ, Buunk AM, et al. Dutch Pharmacogenetics Working Group (DPWG) guideline for the gene-drug interaction of DPYD and fluoropyrimidines. Eur J Hum Genet. 2020;28(4):508-17.

8. Hamzic S, Aebi S, Joerger M, Montemurro M, Ansari M, Amstutz U, et al. Fluoropyrimidine chemotherapy: recommendations for DPYD genotyping and therapeutic drug monitoring of the Swiss Group of Pharmacogenomics and Personalised Therapy. Swiss Med Wkly. 2020;150:w20375.

9. Wörmann B, Bokemeyer C, Burmeister T, Köhne CH, Schwab M, Arnold D, et al. Dihydropyrimidine Dehydrogenase Testing prior to Treatment with 5-Fluorouracil, Capecitabine, and Tegafur: A Consensus Paper. Oncol Res Treat. 2020;43(11):628-36.
