# Supplementary figures and images for: Implementation of dihydropyrimidine dehydrogenase deficiency testing in Europe
Source: ESMO Open. 2023 Mar 28;8(2):101197. doi: 10.1016/j.esmoop.2023.101197 (PMC10163157; doi:10.1016/j.esmoop.2023.101197)

## Genotyping before and after EMA recommendations

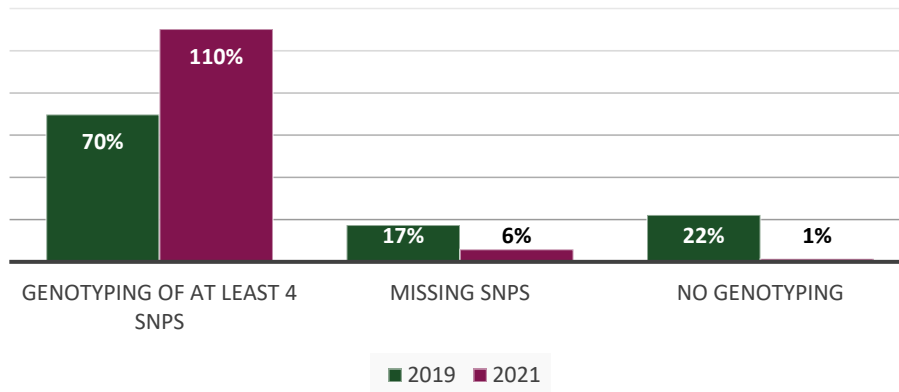

Supplement: Supplementary Figure S1 [file mmc4.pdf]

## Genotyping

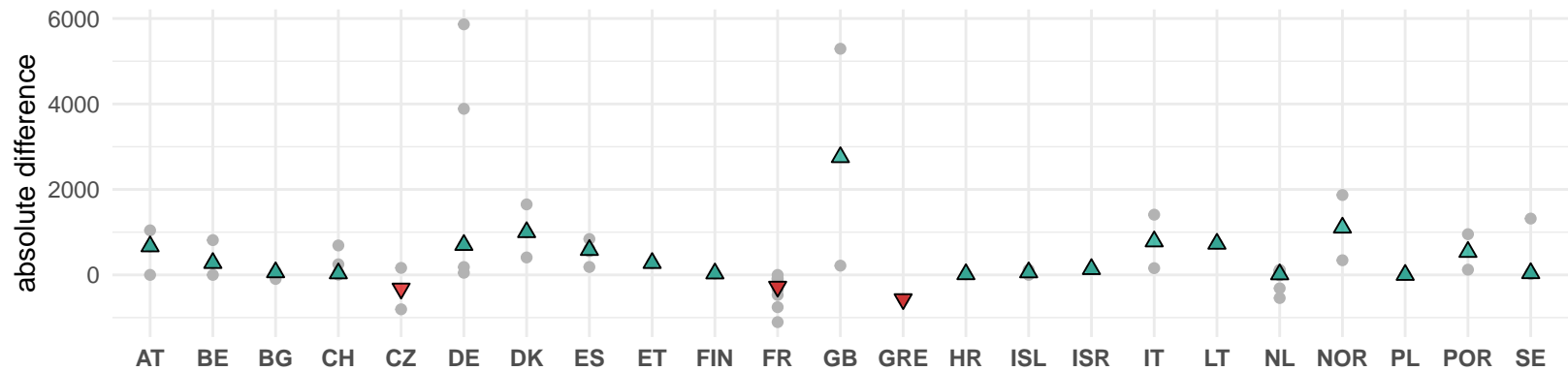

## Phenotyping

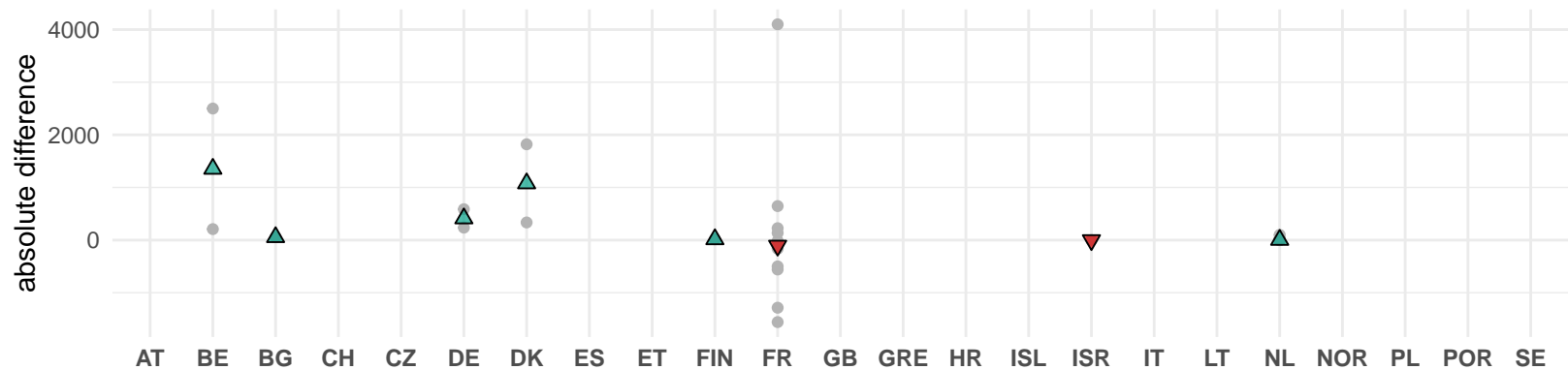

Supplement: Supplementary Figure S2 [file mmc5.pdf]

## A) Genotyping

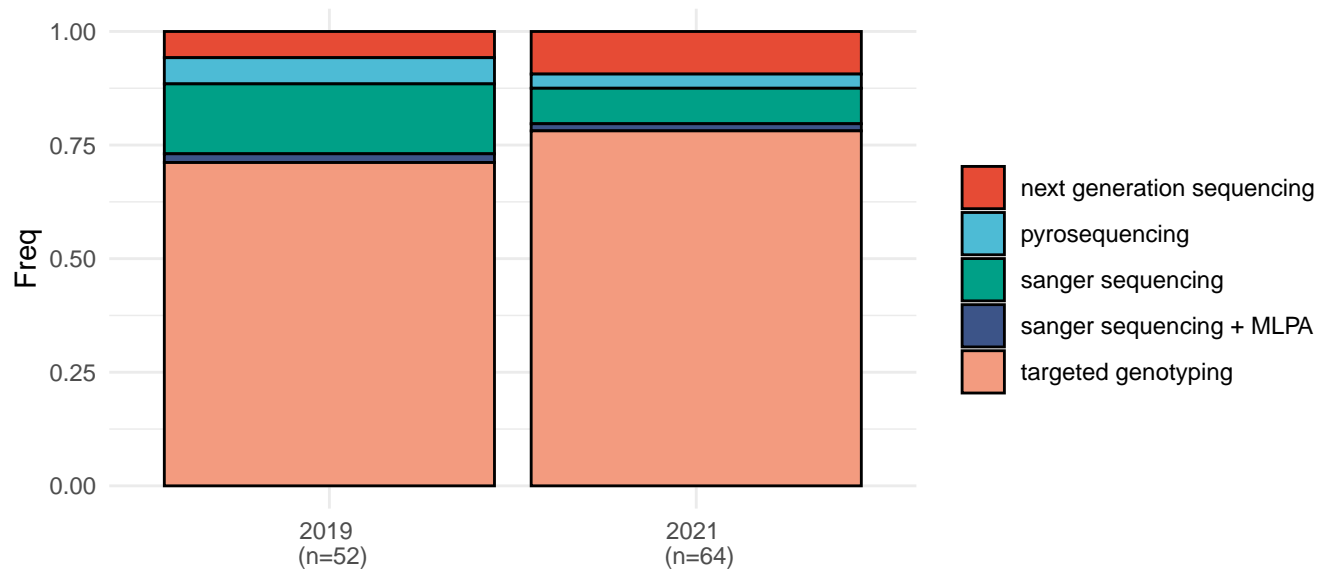

## B) Phenotyping

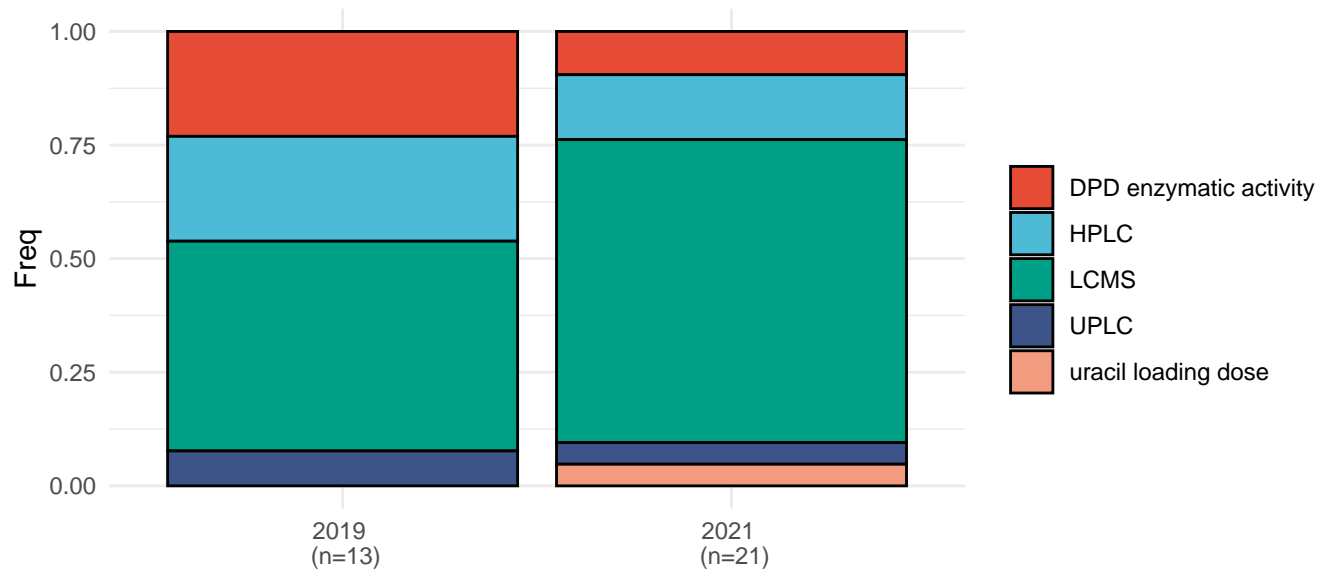

Supplement: Supplementary Figure S4 [file mmc7.pdf]
