# Supplementary material for: Implementation of dihydropyrimidine dehydrogenase deficiency testing in Europe
Source: ESMO Open. 2023 Mar 28;8(2):101197. doi: 10.1016/j.esmoop.2023.101197 (PMC10163157; doi:10.1016/j.esmoop.2023.101197)
Supplement: Supplementary Figure S3 [file mmc6.pptx]

## Slide 1
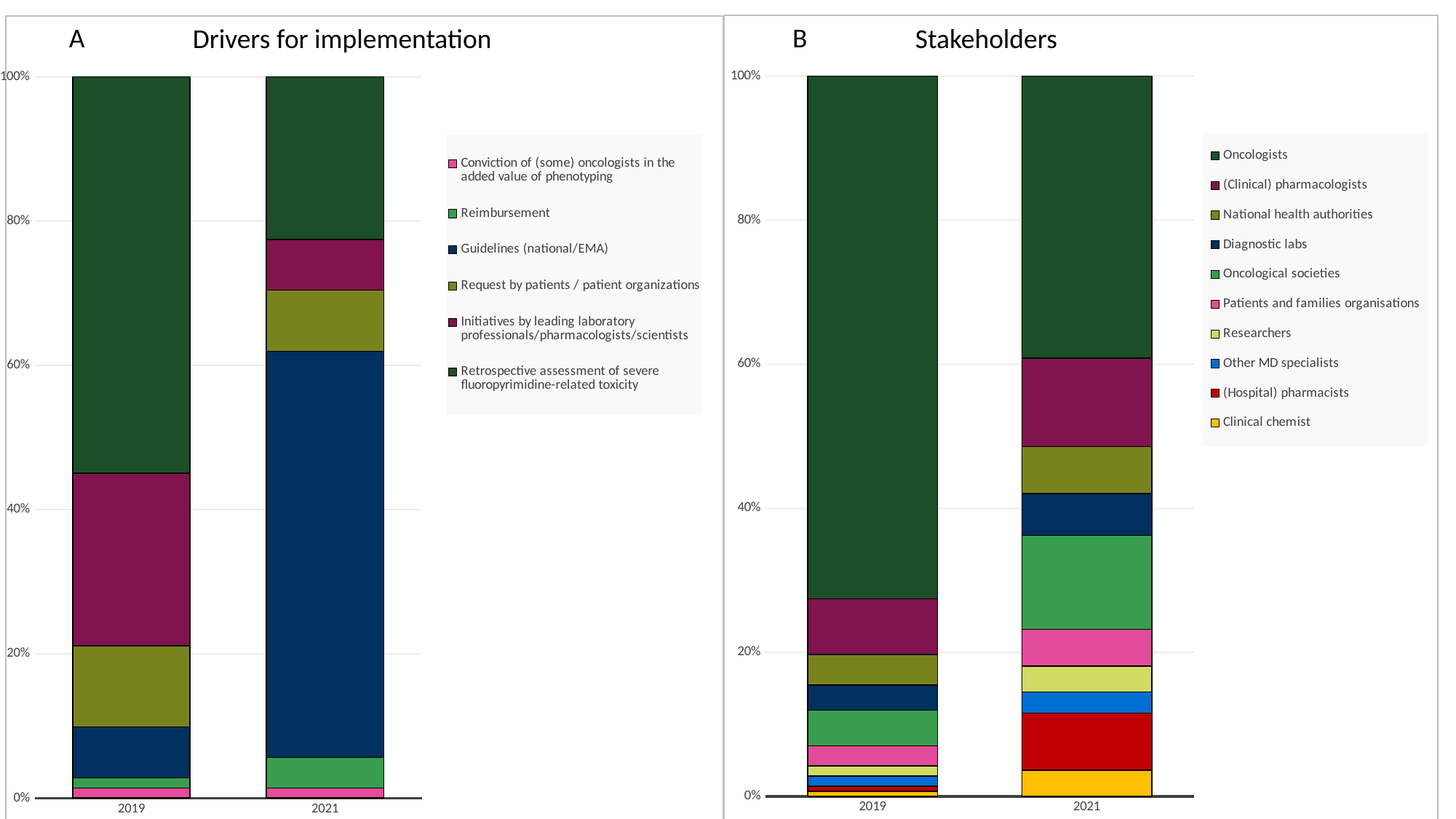

A
### Chart
| Category | Clinical chemist | (Hospital) pharmacists | Other MD specialists | Researchers | Patients and families organisations | Oncological societies | Diagnostic labs | National health authorities | (Clinical) pharmacologists | Oncologists |
|---|---|---|---|---|---|---|---|---|---|---|
| 2019 | 1.0 | 1.0 | 2.0 | 2.0 | 4.0 | 7.0 | 5.0 | 6.0 | 11.0 | 103.0 |
| 2021 | 5.0 | 11.0 | 4.0 | 5.0 | 7.0 | 18.0 | 8.0 | 9.0 | 17.0 | 54.0 |B
### Chart
| Category | Conviction of (some) oncologists in the added value of phenotyping | Reimbursement | Guidelines (national/EMA) | Request by patients / patient organizations | Initiatives by leading laboratory professionals/pharmacologists/scientists | Retrospective assessment of severe fluoropyrimidine-related toxicity |
|---|---|---|---|---|---|---|
| 2019 | 1.0 | 1.0 | 5.0 | 8.0 | 17.0 | 39.0 |
| 2021 | 1.0 | 3.0 | 40.0 | 6.0 | 5.0 | 16.0 |Drivers for implementation
Stakeholders

## Slide 2
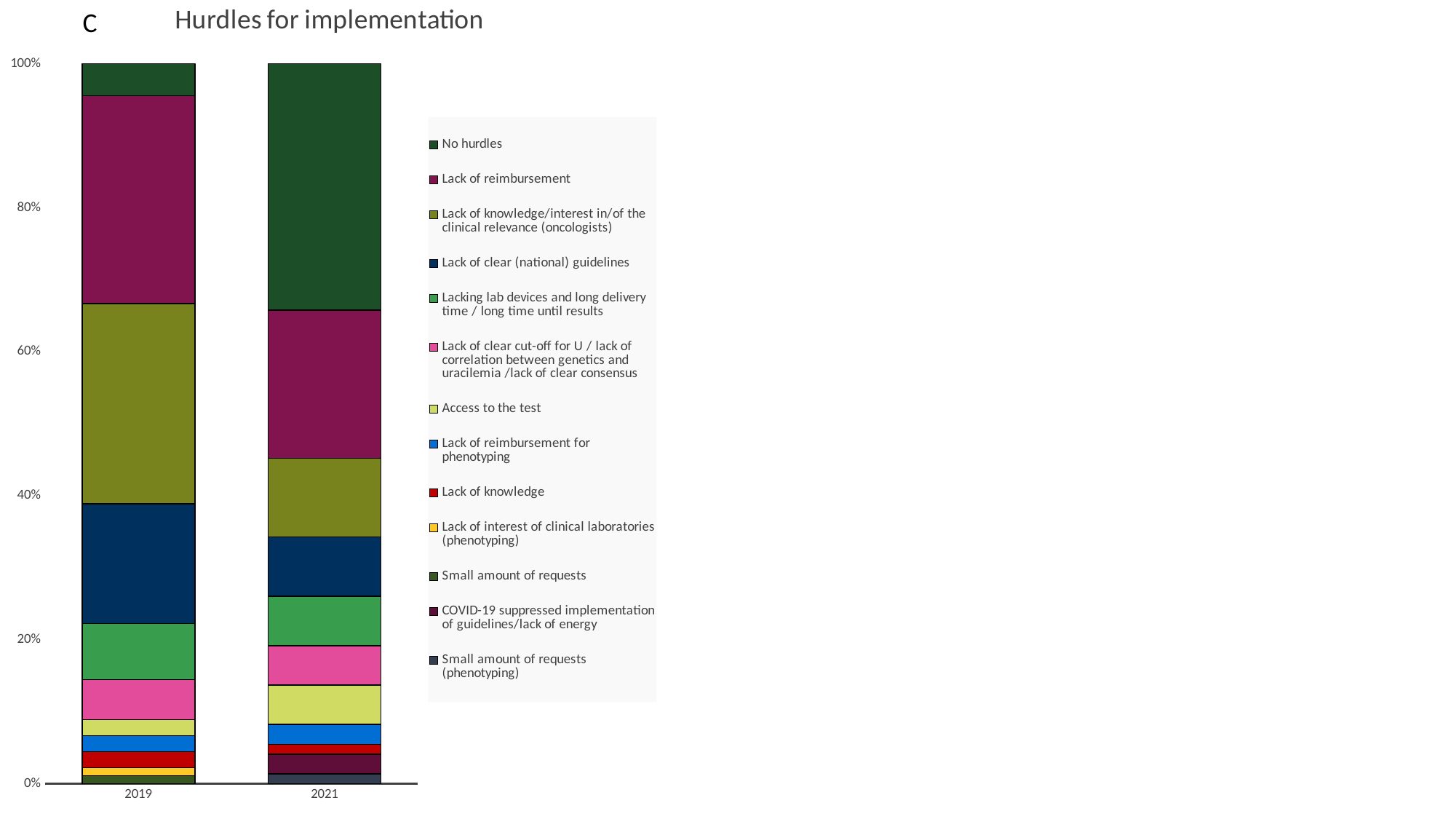

### Chart: Hurdles for implementation
| Category | Small amount of requests (phenotyping) | COVID-19 suppressed implementation of guidelines/lack of energy | Small amount of requests | Lack of interest of clinical laboratories (phenotyping) | Lack of knowledge | Lack of reimbursement for phenotyping | Access to the test | Lack of clear cut-off for U / lack of correlation between genetics and uracilemia /lack of clear consensus | Lacking lab devices and long delivery time / long time until results | Lack of clear (national) guidelines | Lack of knowledge/interest in/of the clinical relevance (oncologists) | Lack of reimbursement | No hurdles |
|---|---|---|---|---|---|---|---|---|---|---|---|---|---|
| 2019 | 0.0 | 0.0 | 1.0 | 1.0 | 2.0 | 2.0 | 2.0 | 5.0 | 7.0 | 15.0 | 25.0 | 26.0 | 4.0 |
| 2021 | 1.0 | 2.0 | 0.0 | 0.0 | 1.0 | 2.0 | 4.0 | 4.0 | 5.0 | 6.0 | 8.0 | 15.0 | 25.0 |C
