# Supplementary material for: Implementation of dihydropyrimidine dehydrogenase deficiency testing in Europe
Source: ESMO Open. 2023 Mar 28;8(2):101197. doi: 10.1016/j.esmoop.2023.101197 (PMC10163157; doi:10.1016/j.esmoop.2023.101197)
Supplement: Supplementary Table S1 [file mmc8.docx]

**Supplementary Table S1**

**Additional variants tested by country in 2019 and 2021.**

| **Country** | **Additional SNPs in 2019** | **Additional SNPs in 2021** |
| --- | --- | --- |
| **Belgium** | PCR and NGS for the complete coding region of *DPYD* | PCR and Sanger sequencing is performed for exons 4, 6, 7, 11, 13, 14, 22, 23 |
| **Bulgaria** | *5 - rs1801159; c.1627A>G ex 13 | *5 - rs1801159; c.1627A>G ex 13  *9A - rs1801265; c.85T>C ex2  *3 - rs72549303; c.1898delC ex14  *4 - rs1801158, c.1601G>A ex 13  *6 - rs1801160; c.2194G>A ex 18  *7 - rs72549309, c.295_298delTCAT ex 4  *8 - rs1801266; c.703C>T ex 7  *10 - rs1801268; c.2983G>T ex 23  *11- rs72549306; c.1003G>T ex10  *12 - rs78060119; c.1156G>T ex 11  *9B - rs1801267b, rs1801265; c.2657G>A, c.85T>C ex2, ex21 |
| **Switzerland** |  | c.557A>G |
| **Czech Republic** | Stop-gain and frame-shift variants and intragenic LGR  rs1801160 | stop-gain and frame-shift variants and intragenic LGR |
| **Germany** |  | c.483+18G>A (rs56276561) |
| **Finland** | Whole genome sequencing + MLPA | Whole genome sequencing + MLPA |
| **France** | All coding exons | All coding exons |
| **Italia** | *DPYD**6 is post-treatment toxicity tested | *DPYD**6 is post-treatment toxicity tested |
| **Netherlands** | c.299_302delTCAT (*7) | c.299_302delTCAT (*7) |
| **Sweden** |  | c.299_302delTCAT (*7) |
| **United Kingdom** | Some centres test for additional SNPs; rs12132152, rs777425216, rs2612091, rs568132506, rs72549309, rs72549308, rs72549307, rs1185250556, rs1801266, DPYD 1039-1042delTG, rs1057516357, rs72549304, rs72549303, rs72547601, rs1801268, rs115232898 | Some centres test for additional SNPs; rs12132152, rs777425216, rs2612091, rs568132506, rs72549309, rs72549308, rs72549307, rs1185250556, rs1801266, DPYD 1039-1042delTG, rs1057516357, rs72549304, rs72549303, rs72547601, rs1801268, rs115232898 |

**Countries are listed in alphabetical order. Abbreviations**: SNPs: Single Nucleotide Polymorphisms.
