# Supplementary material for: Implementation of dihydropyrimidine dehydrogenase deficiency testing in Europe
Source: ESMO Open. 2023 Mar 28;8(2):101197. doi: 10.1016/j.esmoop.2023.101197 (PMC10163157; doi:10.1016/j.esmoop.2023.101197)
Supplement: Supplementary _Materials [file mmc9.docx]

**Supplementary materials:**

**Supplementary Appendix 1**

*Contributors of ‘The European DPD implementation group’.*

**Supplementary Appendix 2**

*Survey used in this study.*

**Supplementary Appendix 3**

*European countries with a guideline regarding DPD deficiency testing, followed by reference.*

**Supplementary Figure S1**

*Genotyping before and after EMA recommendations*

**Supplementary Figure S2**

*Absolute difference in genotyping and phenotyping*

*Absolute difference between 2021 and 2019 in genotyping and phenotyping numbers per country. Responding centers per country are represented as dots, median differences per country are represented as triangle. Countries with only one responding center are Bulgaria (BG), Finland (FIN) and Israel (ISR) for phenotyping, Croatia (HR), Iceland (ISL), Israel (ISR), and Greece (GRE) for genotyping. Absence of point for the phenotyping panel indicates that this testing type is not performed in the country. Countries are ordered alphabetically and labelled following ISO 3166. (Abbreviations: AT = Austria, BE = Belgium, BG = Bulgaria, CH = Switzerland, CZ = Czechia, DE = Germany, DK = Denmark, ES = Spain, ET = Estonia, FIN= Finland, FR = France, GB = Great Britain, GRE = Greece, HR = Croatia, ISL = Iceland, ISR = Israel, IT = Italy, LT = Lithuania, NL = The Netherlands, NOR = Norway, PL = Poland, POR = Portugal, SE = Sweden).*

**Supplementary Figure S3**

*The drivers (A), stakeholders (B), and hurdles (C) for implementation in 2019 (before EMA recommendations) and 2021 (after EMA recommendations).*

**Supplementary Figure S4**

*Genotyping and phenotyping methods in 2019 and 2021*

*Abbreviations: HPLC (high performance liquid chromatography), UPLC (ultra performance liquid chromatography), LCMS (liquid chromatography, mass spectrometry), MLPA (multiplex ligation-dependent probe amplification).*

**Supplementary Table S1**

*Additional variants tested by country in 2019 and 2021*
